# Association between sucrose intake and risk of overweight and obesity in a prospective sub-cohort of the European Prospective Investigation into Cancer in Norfolk (EPIC-Norfolk)

**DOI:** 10.1017/S1368980015000300

**Published:** 2015-02-23

**Authors:** Gunter GC Kuhnle, Natasha Tasevska, Marleen AH Lentjes, Julian L Griffin, Matthew A Sims, Larissa Richardson, Sue M Aspinall, Angela A Mulligan, Robert N Luben, Kay-Tee Khaw

**Affiliations:** 1 Department of Food & Nutritional Sciences, University of Reading, Reading RG6 6AP, UK; 2 Department of Public Health and Primary Care, University of Cambridge, Cambridge, UK; 3 School of Nutrition and Health Promotion, Arizona State University, Phoenix, AZ, USA; 4 Department of Biochemistry, University of Cambridge, Cambridge, UK; 5 MRC Human Nutrition Research Unit, Cambridge, Cambridge, UK; 6 MRC Epidemiology Unit, University of Cambridge, Cambridge, UK

**Keywords:** Obesity, Sugar, Biomarker

## Abstract

**Objective:**

The objective of the present study was to investigate associations between sugar intake and overweight using dietary biomarkers in the Norfolk cohort of the European Prospective Investigation into Cancer and Nutrition (EPIC-Norfolk).

**Design:**

Prospective cohort study.

**Setting:**

EPIC-Norfolk in the UK, recruitment between 1993 and 1997.

**Subjects:**

Men and women (*n* 1734) aged 39–77 years. Sucrose intake was assessed using 7 d diet diaries. Baseline spot urine samples were analysed for sucrose by GC-MS. Sucrose concentration adjusted by specific gravity was used as a biomarker for intake. Regression analyses were used to investigate associations between sucrose intake and risk of BMI>25·0 kg/m^2^ after three years of follow-up.

**Results:**

After three years of follow-up, mean BMI was 26·8 kg/m^2^. Self-reported sucrose intake was significantly positively associated with the biomarker. Associations between the biomarker and BMI were positive (*β*=0·25; 95 % CI 0·08, 0·43), while they were inverse when using self-reported dietary data (*β*=−1·40; 95 % CI −1·81, −0·99). The age- and sex-adjusted OR for BMI>25·0 kg/m^2^ in participants in the fifth *v.* first quintile was 1·54 (95 % CI 1·12, 2·12; *P*_trend_=0·003) when using biomarker and 0·56 (95 % CI 0·40, 0·77; *P*_trend_<0·001) with self-reported dietary data.

**Conclusions:**

Our results suggest that sucrose measured by objective biomarker but not self-reported sucrose intake is positively associated with BMI. Future studies should consider the use of objective biomarkers of sucrose intake.

Obesity and overweight are associated with increased risk for a number of chronic diseases, such as cancer^(^ ^1^ ^)^, CVD^(^ ^2^ ^)^ and type 2 diabetes. However, although energy balance is clearly central, there remains uncertainty about the role of specific dietary factors. While public perception suggests that the intake of sugar is associated with an increased risk of obesity and thus overweight people consume more sugar^(^ ^3^ ^)^, data from observational studies are inconsistent and weight increase or markers of obesity are associated mainly with the intake of sugar-sweetened beverages but not, or only to a small extent, with the total intake of sugar or sucrose^(^ ^4^ ^,^ ^5^ ^)^. Indeed, the US Institute of Medicine reported an inverse association between sugar intake and BMI in adults^(^ ^6^ ^)^. The European Food Safety Authority based its scientific opinion on these findings^(^ ^7^ ^)^, although it omitted acknowledging that the Institute of Medicine considers the finding to be explained mainly by under-reporting. Under-reporting of diet has been found to be more prevalent among women and obese people^(^ ^8^ ^–^ ^10^ ^)^ and it is mainly simple sugars and between-meal snacks that are most commonly under-reported^(11)^. This makes it difficult to interpret the inverse associations between reported sugar intake and BMI and to provide reliable recommendations to the public.

Urinary sugars, in particular sucrose and fructose, have been investigated and developed as dietary biomarkers of total sugar intake^(^ ^12^ ^–^ ^16^ ^)^. If 24 h urine collections are available, sucrose and fructose measured in 24 h urine can be used as predictive biomarkers of total sugar intake^(^ ^16^ ^)^. Given the sugars biomarker is a short-term measure of intake, when measured in spot urine its value will be associated with a certain amount of random error dependent on the timing of the spot urine collection. This error will be expected to attenuate the association between true intake and the biomarker. Nevertheless, earlier work showed sucrose in partial collections to be significantly correlated with sucrose intake^(^ ^13^ ^)^. Previously, we have applied this biomarker to spot urine samples and investigated the association between sugar intake and obesity in a cross-sectional case–control study design in a sub-sample of the Norfolk cohort of the European Prospective Investigation into Cancer and Nutrition (EPIC-Norfolk), which only included normal weight (BMI≤25·0 kg/m^2^) and obese (BMI ≥ 30·0 kg/m^2^) participants^(^ ^3^ ^)^. In that study we found a significant positive association between the biomarker and obesity (OR=2·44; 95 % CI 1·54, 3·86 for the bottom *v.* top quintile).

Here, we prospectively investigated the association between sucrose intake and risk of overweight and obesity in a sample of the EPIC-Norfolk cohort study using urinary sugar biomarkers and self-reported dietary data.

## Materials and methods

### Study population

Between 1993 and 1997, approximately 77 630 healthy men and women were invited to participate in the EPIC-Norfolk study through thirty-five medical practices in Norfolk^(^ ^17^ ^,^ ^18^ ^)^; 25 639 participants, aged between 39 and 79 years, agreed to participate and attended the first health examination. Diet was assessed by 7 d diet diary (7DD) and a 130-item semi-quantitative FFQ. The first day of the diary was completed as a 24 h recall (24HDR) with a trained interviewer, whereas the remainder was completed during subsequent days by the participants at home. Diary data were processed using the in-house dietary assessment software DINER (Data Into Nutrients for Epidemiological Research)^(^ ^19^ ^)^; data were checked and calculated using DINERMO, the software used to process data entered by DINER^(^ ^20^ ^)^. FFQ data were analysed using the in-house program, FETA (FFQ EPIC Tool for Analysis), to calculate the nutrient content^(^ ^21^ ^)^. Health and lifestyle characteristics were assessed by a questionnaire. Physical activity, representing occupational and leisure activity, was assessed using a validated questionnaire^(^ ^22^ ^)^, using four categorical variables (inactive, moderately inactive, moderately active and active). Height and weight measurements were collected following a standardised protocol as part of a health check conducted by trained research nurses^(^ ^23^ ^)^. Spot urine samples were collected at baseline during the study visit (day 2 of the diary) and stored at −20°C without preservatives. The study received ethical approval by the Norwich District Health Authority Ethics Committee and all participants gave signed informed consent.

Participants were invited back for a second health examination after three years of follow-up from 1997 to 2000 and 15 786 participants attended. A health and lifestyle questionnaire was completed before the health examination. At the health examination, the protocol of the first health examination was repeated and data on height, weight and waist circumference (WC) were collected by trained nurses. The anthropometric measures collected at the second health examination were used as outcome measures in the analysis.

### Sample selection and missing data

**Fig. 1:**
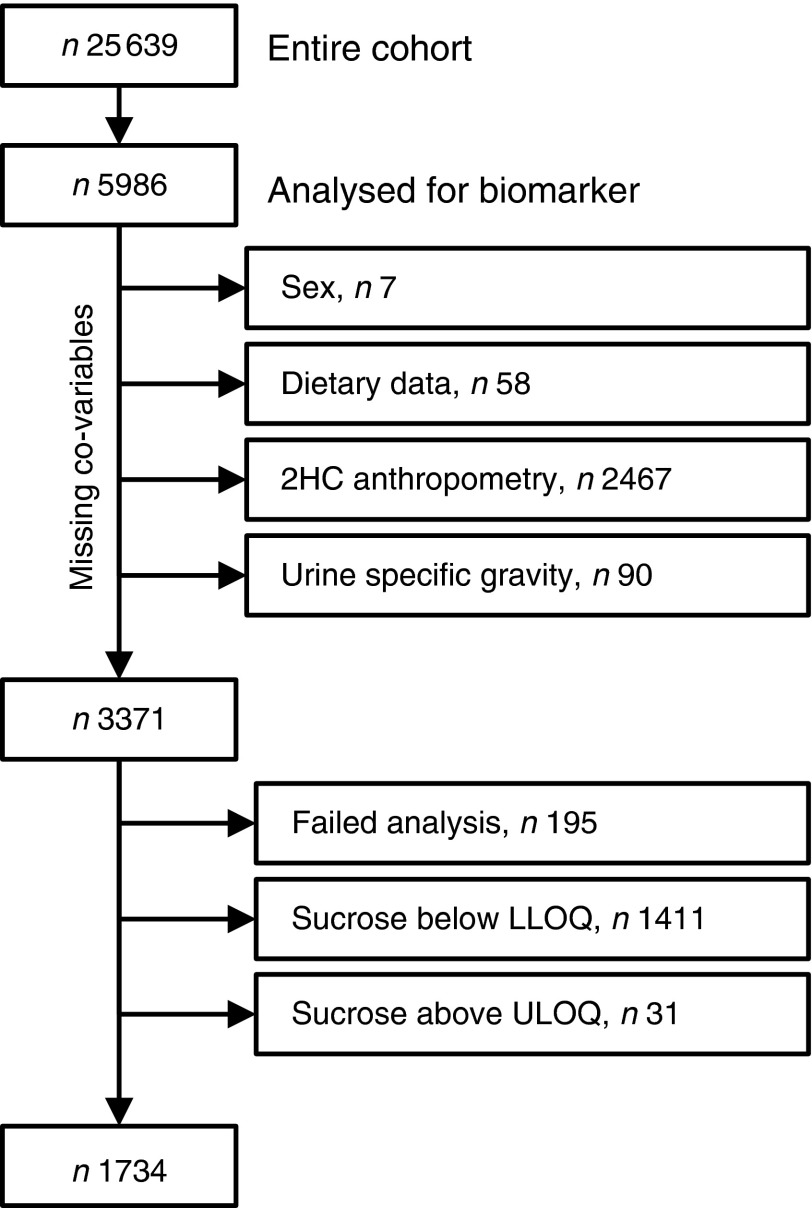
Study population and sample size (2HC, second health check; LLOQ, lower limit of quantification; ULOQ, upper limit of quantification)

Baseline spot urine samples (*n*5993) were selected randomly from the storage facility. While this selection was random, the samples are not necessarily a representative selection of the cohort. Co-variables (sex, dietary data and specific gravity) were missing for 155 participants and end points (anthropometric data) were missing for a further 2467 who did not attend the second health examination. Urinary sucrose analyses failed for 195 participants and results were outside the calibration range for 1442 participants, leaving a total sample size of 1734 (see Fig. 1 for details). For sensitivity analyses, sucrose concentrations below and above the limits of quantification (<5·0 µm or>500 µm) were imputed with 4·9 µm and 500·1 µm, respectively.

### Analytical method

Urinary sucrose and fructose were analysed using a modified version of the method described previously^(^ ^24^ ^)^. Urine aliquots (200 µl) were mixed with 50 µl internal standard solution ([^13^C_6_]fructose and [^13^C_12_]sucrose, 100 µm in water; CK Isotopes, Ibstock, UK) and 800 µl cold acetonitrile was added to precipitate proteins. Samples were processed using a Hamilton Star (HAMILTON Robotics Ltd, Birmingham, UK) robot. The samples were mixed and centrifuged for 30 min at 14 000***g***, then 500 µl of the supernatant was transferred into a silanised glass vial and dried under reduced pressure. The samples were then derivatised as described previously^(^ ^24^ ^,^ ^25^ ^)^. Briefly, the samples were reconstituted in 30 µl methoxyamine hydrochloride (20 mg/ml in dry pyridine; Sigma-Aldrich, Gillingham, UK), mixed and incubated at room temperature for 30 min. After 16 h at room temperature, 30 µl *N*-methyl-*N*-trimethylsilyl-trifluoroacetamide containing 1 % trimethylchlorosilane (Sigma-Aldrich) was added to each sample and incubated for 30 min at 75°C. The derivatised samples were diluted with 540 µl dry acetonitrile.

Samples were then analysed with a Trace GC Ultra and a Trace DSQ quadrupole mass spectrometer (ThermoElectron, Hemel Hempstead, UK). The derivatised sample was injected with a 1:10 split on to a 30 m×0·25 mm i.d., 5 % phenylpolysilphenylene-siloxane column with a chemically bonded 25 mm TR-5MS stationary phase (ThermoElectron). The oven temperature was kept at 60°C for 2 min and then increased by 58°C/min to 310°C. The carrier gas was helium (flow rate 1·2 ml/min). The mass spectrometer (transfer line temperature: 250°C; ion source temperature: 275°C; electron beam: 70 eV) was operated in full scan mode (50–650 *m*/*z*; 3 scans/s) and compounds were identified by their retention time and characteristic fragments.

Samples were quantified using the peak area ratio (analyte:internal standard) using an eight-point calibration line with samples prepared in water (concentration in µm: 5, 10, 15, 25, 50, 75, 100 and 150). The difference between back-calculated and actual concentrations was always less than 5 %. Samples with a concentration outside the calibration range were diluted and re-analysed. Fructose was quantified using the sum of the peak area of the two epimers of the analyte and the internal standard. Quality control samples were prepared by adding known amounts of sucrose and fructose to spot urine samples and including at least three (with low, medium and high concentrations) in each analytical batch. Supplemental Table 1 (see online supplementary material) shows the reproducibility of the method for urinary sucrose and fructose at different concentrations. Urinary sucrose and fructose concentration remained stable for at least 7 d at 4°C.

**Table 1:** Associations between sucrose intake (by biomarker or 7 d diet diary (7DD)), BMI and waist circumference (WC) after three years of follow-up at the second health check among men and women (*n* 1734) aged 39–77 years, Norfolk cohort of the European Prospective Investigation into Cancer and Nutrition (EPIC-Norfolk)

	Sucrose intake and biomarker in separate models						Sucrose intake and biomarker in the same model					
	BMI (kg/m^2^) adjusted for age			WC (cm) adjusted for age and height			BMI (kg/m^2^) adjusted for age			WC (cm) adjusted for age and height		
	*β*	95 % CI	*P*	*β*	95 % CI	*P*	*β*	95 % CI	*P*	*β*	95 % CI	*P*
Men (*n* 797)												
Biomarker*	0·09	−0·13, 0·31	0·442	0·42	−0·22, 1·06	0·195	0·22	0·00, 0·45	0·052	0·82	0·17, 1·46	0·013
Intake†	−1·18	−1·67, −0·69	<0·001	−3·35	−4·78, −1·93	<0·001	−1·30	−1·81, −0·79	<0·001	−3·79	−5·25, −2·33	<0·001
Women (*n* 937)												
Biomarker*	0·40	0·14, 0·65	0·002	0·85	0·24, 1·46	0·007	0·50	0·25, 0·76	<0·001	1·12	0·52, 1·73	<0·001
Intake†	−1·60	−2·25, −0·96	<0·001	−4·19	−5·75, −2·64	<0·001	−1·80	−2·45, −1·15	<0·001	−4·63	−6·19, −3·07	<0·001
Sex-adjusted (*n* 1734)												
Biomarker*	0·25	0·08, 0·43	0·004	–		–	0·38	0·21, 0·55	<0·001	–		–
Intake†	−1·40	−1·81, −0·99	<0·001	–		–	−1·57	−1·99, 1·16	<0·001	–		–

Data are shown with biomarker and intake in separate models as well as in the same model. Regression coefficients *β* and 95 % confidence intervals were determined by linear regression. Biomarker and intake data were log-transformed before analysis.

* Urinary sucrose, adjusted by specific gravity.

† 7DD sucrose intake, energy-adjusted (g/MJ).

The STROBE (Strengthening the Reporting of Observational Studies in Epidemiology) checklist for cohort studies^(^ ^26^ ^)^ has been completed for the present study.

### Data analysis

For data analyses, samples with urinary sucrose concentrations outside the acceptable quantification range were excluded. Based on the data shown in Supplemental Table 1, the acceptable range for sucrose was 5 µm to 500 µm (1·7 mg/l to 171 mg/l) and 20 µm to 500 µm (3·6 mg/l to 90 mg/l) for fructose. Data on urinary fructose were used in the sensitivity analysis only, whereas the main analysis was conducted using urinary sucrose as a biomarker for sucrose intake. Concentration of sucrose in urine was expressed relative to specific gravity to adjust for urine concentration. Creatinine could not be used, given the highly significant association between urinary creatinine and BMI (*ρ*=0·15; *P*<0·0001).

The distributions of the biomarker (urinary sucrose) and self-reported sugar intake were skewed and therefore all analyses with continuous data were conducted with log-transformed data. Both sex-specific and sex-adjusted analyses were conducted. 7DD sucrose intake was adjusted for energy intake using the nutrient density method (g/MJ). The association of self-reported energy-adjusted sucrose intake or sucrose biomarker with BMI and WC at the second health examination was assessed using linear regression models adjusted for age and sex; for WC, the model was also adjusted for height at the second health check. We report *β* coefficients for the regression of BMI or WC *v*. log-transformed biomarker or self-reported energy-adjusted sucrose intake. Participants were divided into quintiles by urinary biomarker (sucrose adjusted by specific gravity) and dietary intake (self-reported sucrose intake, 7DD). Odds ratios and 95 % confidence intervals for BMI>25·0 kg/m^2^ after three years of follow-up were estimated using unconditional logistic regression in age- and sex-adjusted models. Tests for linear trend were conducted by treating quintiles as continuous variables.

We also included biomarker (log-transformed) and self-reported intake (7DD, energy-adjusted, log-transformed) in the same linear regression model to calculate adjusted means of BMI (additionally adjusted for age) and WC (additionally adjusted for age and height) after three years of follow-up.

Statistical analyses were conducted with the statistical software package Stata version 11·2 and R^(^ ^27^ ^)^. The *P* values for statistical tests were two-tailed and considered statistically significant at a level of less than 0·05.

## Results

### Study population

Spot urine samples were analysed from a selection of 5986 participants (2578 men, 43 %; 3408 women, 57 %) of EPIC-Norfolk. Co-variables and end points were available for 3371 participants (1338 men, 41 %; 1983 women, 59 %; Fig. 1). Urinary sucrose concentration was outside the calibration range (5 µm to 500 µm) or could not be detected due to analytical problems in 1637 participants, leaving 1734 participants (797 men, 46 %; 937 women, 54 %) for whom biomarker data were available. After three years of follow-up, the mean BMI increased from 26·2 (95 % CI 26·0, 26·4) kg/m^2^ at baseline to (95 % CI 26·6, 27·0) 26·8 kg/m^2^, and 35 %, 48 % and 17 % of the participants were normal weight, overweight and obese, respectively. The mean WC was 96·9 (95 % CI 96·2, 97·6) cm in men and 82·1 (95 % CI 81·5, 82·8) cm in women.

We compared urinary sucrose, adjusted by specific gravity, as a biomarker for sucrose intake with energy-adjusted, self-reported 7DD sucrose intake. Supplemental Table 2 gives a summary of the baseline characteristics of study participants, divided into quintiles by biomarker and self-reported intake, respectively (more details are shown in Supplemental Tables 3 and 4, see online supplementary material). Independent of the classification method used (biomarker or energy-adjusted self-reported intake), age, energy and sucrose intake (g/d) increased across quintiles. The proportion of dietary sucrose to total dietary sugars also increased from the bottom to the top quintile, with a larger range being observed when using self-reported dietary data. However, while there were more women in the bottom quintile of biomarker and more men in the top one, this relationship was reversed when using self-reported dietary data. Mean BMI measured at both baseline and the second health check increased across quintiles for the biomarker, while it decreased across quintiles for self-reported sucrose. Similar observations were made for WC measured at the second health check.

**Table 2:** Associations between sucrose intake and risk of being overweight or obese after three years of follow-up at the second health check among men and women (*n* 1734) aged 39–77 years, Norfolk cohort of the European Prospective Investigation into Cancer and Nutrition (EPIC-Norfolk)

	Men (*n* 797)					Women (*n* 937)					All (*n* 1734)				
	Overweight*					Overweight*					Overweight*				
	*n*	%	OR	95 % CI	*P*	*n*	%	OR	95 % CI	*P*	*n*	%	OR	95 % CI	*P*
Urinary sucrose, adjusted by specific gravity															
Q1	105	66	1·00	Ref.	–	106	56	1·00	Ref.	–	201	58	1·00	Ref.	–
Q2	114	72	1·31	0·82, 2·11	0·263	98	52	0·84	0·56, 1·27	0·416	216	62	1·15	0·84, 1·56	0·384
Q3	115	72	1·33	0·83, 2·14	0·238	105	56	0·96	0·64, 1·45	0·857	225	65	1·26	0·92, 1·71	0·149
Q4	119	75	1·54	0·95, 2·50	0·082	123	66	1·45	0·96, 2·21	0·080	237	68	1·43	1·05, 1·96	0·025
Q5	120	75	1·57	0·96, 2·57	0·070	119	63	1·33	0·88, 2·02	0·180	245	71	1·54	1·12, 2·12	0·008
Trend	–		1·11	1·00, 1·25	0·054	–		1·12	1·02, 1·23	0·020	–		1·12	1·04, 1·20	0·003
Sucrose intake, 7DD energy-adjusted															
Q1	128	80	1·00	Ref.	–	120	64	1·00	Ref.	–	255	73	1·00	Ref.	–
Q2	123	77	0·84	0·49, 1·44	0·525	117	63	0·92	0·61, 1·41	0·710	236	68	0·77	0·56, 1·08	0·129
Q3	106	66	0·48	0·29, 0·80	0·005	107	57	0·73	0·48, 1·11	0·146	209	60	0·56	0·41, 0·78	0·001
Q4	107	67	0·50	0·30, 0·84	0·008	103	55	0·68	0·45, 1·03	0·068	212	61	0·58	0·42, 0·80	0·001
Q5	109	69	0·53	0·32, 0·89	0·015	104	56	0·69	0·45, 1·04	0·076	212	61	0·56	0·40, 0·77	<0·001
Trend	–		0·84	0·75, 0·94	0·003	–		0·90	0·82, 0·99	0·026	–		0·87	0·81, 0·93	<0·001

7DD, 7 d diet diary; Q1, quintile 1 (lowest); Q2, quintile 2; Q3, quintile 3; Q4, quintile 4; Q5, quintile 5 (highest); Ref., referent category. Odds ratios and 95 % confidence intervals were determined by logistic regression, adjusted for age and sex.

* Second health check, BMI>25·0 kg/m^2^.

**Table 3:** Comparison of different assessment methods: associations (regression coefficients *β* and 95 % confidence intervals) between sucrose intake (log-transformed) and BMI and waist circumference (WC) after three years of follow-up among men and women (*n* 1734) aged 39–77 years, Norfolk cohort of the European Prospective Investigation into Cancer and Nutrition (EPIC-Norfolk)

	BMI (kg/m^2^)*,†		WC (cm)*,‡			
	All		Men		Women	
	*β*	95 % CI	*β*	95 % CI	*β*	95 % CI
7DD (adjusted for energy intake)	−1·40	−1·81, −0·99	−3·34	−4·77, −1·91	−4·21	−5·77, −2·66
24HDR§ (adjusted for energy intake)	−0·78	−1·06, −0·51	−2·31	−3·33, −1·30	−2·14	−3·13, −1·15
FFQ (adjusted for energy intake)	−1·32	−1·83, −0·81	−2·74	−4·54, −0·94	−2·52	−4·44, −0·59
7DD	−1·38	−1·72, −1·04	−2·80	−4·00, −1·61	−3·85	−5·14, −2·56
24HDR	−0·80	−1·04, −0·57	−2·03	−2·92, −1·13	−2·05	−·288, −1·22
FFQ	−1·07	−1·42, −0·71	−2·76	−4·03, −1·49	−1·87	−3·19, −0·54
Biomarker||	0·25	0·08, 0·43	0·43	−0·21, 1·06	0·87	0·26, 1·49

7DD, 7 d diet diary; 24HDR, 24 h recall.

* At second health check.

† Adjusted for age and sex.

‡ Adjusted for height and age.

§ Among 1685 participants only.

|| Urinary sucrose adjusted by specific gravity.

**Table 4:** Main sources of sucrose intake in men (*n* 797) and women (*n* 937) aged 39–77 years, Norfolk cohort of the European Prospective Investigation into Cancer and Nutrition (EPIC-Norfolk), based on data from 7 d diaries

	Mean	sd	Contribution (%)
Men			
Table sugar	16·8	25·3	29
Cake	8·9	9·8	15
Fruits	6·2	5·9	11
Sweet biscuits	3·7	4·9	6
Pudding (milk-based)	2·8	3·6	5
Confectionery (chocolate)	2·8	5·4	5
Breakfast cereals	2·0	3·6	3
Jam and marmalade	1·7	3·8	3
Squash and lemonade	1·5	4·4	3
Pudding (cereal-based)	1·5	3·7	3
Women			
Fruits	7·5	5·8	18
Table sugar	6·5	14·1	15
Cake	6·3	6·8	15
Confectionery (chocolate)	2·9	5·0	7
Sweet biscuits	2·8	3·4	7
Pudding (milk-based)	2·3	3·0	5
Yoghurt	1·8	3·2	4
Breakfast cereals	1·6	2·6	4
Confectionery (non-chocolate)	1·4	3·5	3
Juices	1·4	2·3	3

Mean and standard deviation of intake in g/d and percentage contribution to total sucrose intake.

### Association between intake and biomarker

In sex-combined analysis, energy-adjusted intake of sucrose (7DD) was significantly associated with the biomarker (*β*=0·078; 95 % CI 0·059, 0·097) and this association did not change materially when adjusted for age and sex. In men, the association was considerably stronger (*β*=0·108; 95 % CI 0·079, 0·138) than in women (*β*=0·060; 95 % CI 0·035, 0·085). This association was also significant for non-energy adjusted sucrose intake (*β*=0·188; 95 % CI 0·144, 0·232; per 25 g/d increase in sucrose intake) and remained stable after adjusting for age and sex. As above, the association was stronger in men (*β*=0·231; 95 % CI 0·155, 0·306; per 25 g/d increase in sucrose intake) than in women (*β*=0·094; 95 % CI 0·045, 0·143; per 25 g/d increase in sucrose intake).

**Fig. 2:**
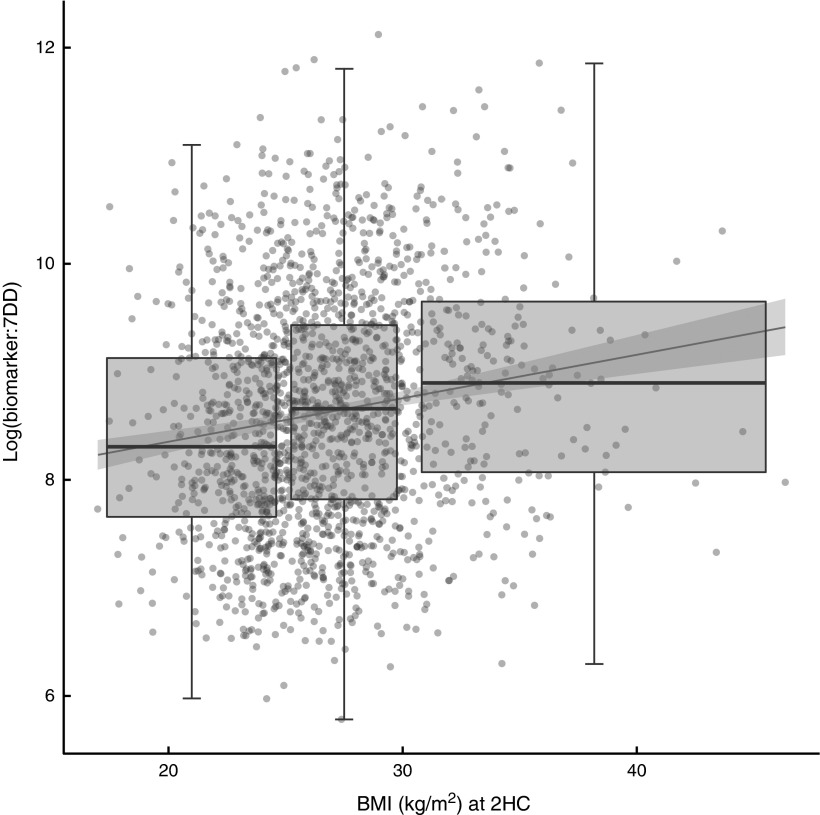
Relationship between the biomarker (specific-gravity-adjusted urinary sucrose) and self-reported intake (energy-adjusted sucrose intake, as assessed by 7 d diet diary (7DD)), expressed as a ratio, and BMI after three years of follow-up among men and women (*n* 1734) aged 39–77 years, Norfolk cohort of the European Prospective Investigation into Cancer and Nutrition (EPIC-Norfolk). Presented are box-and whisker plots in which the bottom and top of the box represents the 25th and 75th percentile, respectively (the interquartile range), the line within the box represents the median and the bottom and top of the whisker represents the minimum and maximum value, respectively, of log-transformed ratio of biomarker to 7DD for three BMI classes (normal weight (left), overweight (middle) and obese (right)) at the second health check (2HC); and a least-square linear model with 95 % confidence interval (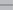)

The relationship between biomarker and self-reported intake, expressed as the ratio of biomarker to energy-adjusted sucrose intake, was positively associated with baseline and follow-up BMI (Fig. 2). Indeed, there was a significant positive association with BMI at follow-up (*β*=0·04; 95 % CI 0·03, 0·05) in an unadjusted model and after adjusting for age and sex (*β*=0·04; 95 % CI 0·02, 0·05). The median ratio was approximately 50 % higher in overweight and obese participants when compared with normal-weight participants.

### Associations between self-reported sucrose intake, biomarker, BMI and waist circumference

Table 1 shows the associations between sucrose, determined either by biomarker or 7DD, and BMI and WC at follow-up. The data show a positive association for the biomarker with BMI and WC, but an inverse association for both energy-adjusted (*β*=−1·40; 95 % CI −1·81, −0·99) and absolute (*β*=−1·38; 95 % CI −1·72, −1·04) self-reported intake (adjusted for age and sex). These associations were statistically significant for women with both biomarker and 7DD data, but only with 7DD data for men. When including biomarker and self-reported dietary data in the same model, the associations were strengthened and remained in opposite directions (biomarker, log-transformed: *β*=0·38; 95 % CI 0·21, 0·55; dietary data, energy-adjusted and log-transformed: *β*=−1·57; 95 % CI −1·99, −1·16; Table 1). Similar relationships were found after stratification by sex (data not shown).

### Associations between self-reported sucrose intake, biomarker and risk of being overweight

**Fig. 3:**
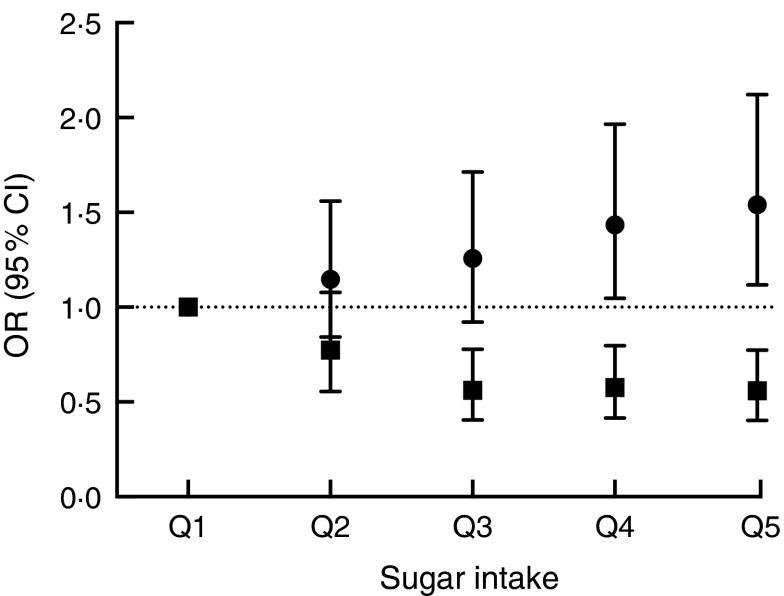
Association between sucrose intake and risk of overweight or obesity after three years of follow-up using either dietary data (■, energy-adjusted, as assessed by 7 d diet diary) or biomarker (●, urinary sucrose, adjusted by specific gravity) among men and women (*n* 1734) aged 39–77 years, Norfolk cohort of the European Prospective Investigation into Cancer and Nutrition (EPIC-Norfolk). Presented are odds ratios with their 95 % confidence intervals represented by vertical bars (Q1, quintile 1 (lowest); Q2, quintile 2; Q3, quintile 3; Q4, quintile 4; Q5, quintile 5 (highest))

There were significant associations between the biomarker and risk of being overweight or obese after three years of follow-up (Table 2, Fig. 3) with an OR of 1·54 (95 % CI 1·12, 2·12; *P*=0·008) between the top and bottom quintile and a significant trend (*P*=0·003) across quintiles. Stratification by sex showed a marginally non-significant trend (*P*=0·054) in men and a significant (*P*=0·02) trend in women. Conversely, there was an inverse association when using self-reported intake with an OR of 0·56 (95 % CI 0·40, 0·77; *P*<0·0001) and also a significant trend (*P*<0·0001). After stratification for sex, the trend remained significant in both men and women. When using the biomarker as a continuous variable, the OR for BMI>25·0 kg/m^2^ was 1·16 (95 % CI 1·05, 1·27) per log increase. Conversely, the OR was 0·60 (95 % CI 0·47, 0·77) when using self-reported dietary data. An analysis of the association between intake and risk of being overweight or obese at baseline gave similar results in the sex-adjusted model (see online supplementary material, Supplemental Table 5).

The ratio of urinary sucrose to fructose concentration has been used previously as a biomarker of sugar intake in relation to risk of being overweight^(^ ^3^ ^)^. Applying this biomarker in the current study reduced the sample size, given there were fewer available samples with both sucrose and fructose values within the acceptable analytical range, to 1238 participants (578 men, 47 %; 660 women, 53 %). The associations observed with urinary sucrose to fructose ratio were not materially different from those observed with specific-gravity adjusted sucrose concentration in urine (see online supplementary material, Supplemental Table 6).

### Comparison of different dietary assessment instruments (7 d diet diary, 24 h recall and FFQ)

Energy-adjusted sucrose intake by 24HDR (first day of 7DD) was significantly associated with estimates from 7DD (*β*=1·08; 95 % CI 1·04, 1·13) and the biomarker (*β*=0·11; 95 % CI 0·08, 0·14; age- and sex-adjusted model). The latter association was stronger in men than in women (data not shown).

FFQ-measured sucrose (g/MJ), available for 1685 participants, was significantly associated with sucrose measured by both 7DD (*β*=0·70; 95 % CI 0·65, 0·75) and 24HDR (*β*=0·88; 95 % CI 0·81, 0·96). The association with the biomarker was also significant (*β*=0·05; 95 % CI 0·04, 0·07; age- and sex-adjusted model) and stronger in men than in women.

Self-reported sucrose intake, independent of the dietary assessment instrument used and adjustment for energy intake, was inversely associated with BMI and WC after three years of follow-up (Table 3), although the associations were weakest with data from 24HDR.

### Effect of physical activity

The associations observed did not change materially after including physical activity in the model (see online supplementary material, Supplemental Table 7).

### Sensitivity analyses with biomarker data outside the calibration range

Biomarker data were available for 3176 participants (1297, 41 % men; 1879, 59 % women; see online supplementary material, Supplemental Table 8 for details), although the urinary sucrose concentration in 1411 participants was below and in thirty-one participants was above the limit of quantification. In men, baseline BMI of participants with urinary sucrose concentration below the limit of quantification (*n* 498; mean BMI=26·2 kg/m^2^) was significantly (*P*=0·04, *t* test) lower than that of participants with sucrose concentration within the calibration range (*n* 821; mean BMI=26·5 kg/m^2^). No significant difference was observed in women (*P*=0·65).

After imputation of data below and above the calibration range with 4·9 µm and 500·1 µm respectively, the urinary sucrose biomarker was significantly positively associated with self-reported sucrose intake (*β*=0·051; 95 % CI 0·039, 0·063). The associations with BMI at follow-up (*β*=0·17; 95 % CI 0·06, 0·27; adjusted for age and sex) and WC at follow-up up (men: *β*=0·55; 95 % CI 0·15, 0·95; women: *β*=0·54; 95 % CI 0·15, 0·93; both adjusted for age) were also significant. The association between the biomarker and risk of being overweight was also significant, with an OR of 1·08 (95 % CI 1·01, 1·14; adjusted for age and sex) per log increase of biomarker; the OR when using dietary data was 0·61 (95 % CI 0·51, 0·72; adjusted for age and sex).

### Sensitivity analysis with gastric ulcer status

A diagnosis of gastric ulcer was reported by sixty-seven participants (thirty-seven men, thirty women) and they did not differ significantly in age and BMI (baseline and second health check). Urinary sucrose concentration was significantly different in men (two-sided *t* test with log-transformed data, geometric mean (SD); gastric ulcer *v.* other: 53 (sd 1) µm *v.* 36 (sd 1) µm; *P*=0·04) but not in women (26 (sd 1) µm *v.* 27 (sd 1) µm; *P*=0·8). Participants with self-reported gastric ulcer were not equally distributed across quintiles of biomarker (*P*=0·004, *χ* ^2^ test), with most participants found in the fourth (*n* 22), fifth (*n* 18) and first (*n* 14) quintiles.

The associations between biomarker and self-reported energy-adjusted sucrose intake, BMI and WC at the second health check were not materially different after excluding participants with self-reported gastric ulcer (see online supplementary material, Supplemental Table 9). The estimated risk of being overweight or obese after follow-up was slightly attenuated, yet the results were not materially different (see online supplementary material, Supplemental Table 10).

## Discussion

In the present study, we investigated prospectively the risk of overweight and obesity in relation to sucrose intake estimated by 7DD and biomarker. Using urinary sucrose as the measure of sucrose intake, participants in the highest *v*. the lowest quintile for sucrose intake had 54 % greater risk of being overweight or obese. In contrast, using self-reported sucrose intake measured by 7DD, those in the highest *v*. the lowest quintile for self-reported sucrose intake were at 44 % lower risk of being overweight or obese.

Our results show a clear discrepancy in the association between sucrose intake and the risk of overweight or obesity depending on the dietary assessment method. The associations observed with self-reported intake are in agreement with data from cross-sectional studies published previously^(^ ^3^ ^,^ ^28^ ^)^, in which a cross-sectional analysis of 875 participants of EPIC-Norfolk found a strong positive association between sugar intake and obesity risk when using the biomarker (trend per quintile, normal weight *v.* obese: OR=1·26; 95 % CI 1·14, 1·40; *P*<0·0001)^(^ ^3^ ^)^.

There are several possible reasons for this apparent discrepancy. The inverse association between self-reported sucrose intake and body weight has been used as a basis for dietary recommendations^(^ ^7^ ^)^. First the suitability of the biomarker used in the present study needs consideration: while there is only limited information on the physiological mechanisms underlying the absorption and excretion of sucrose, several controlled-feeding studies have shown a clear dose–response relationship between sucrose intake and urinary excretion^(^ ^12^ ^–^ ^14^ ^)^, which is independent of BMI^(^ ^15^ ^)^. This has led to the development and validation of 24 h urinary sucrose and fructose as a biomarker for total sugar intake^(^ ^16^ ^)^. However, 24 h urine samples are difficult to obtain and are cost-prohibitive in large observational studies, such as EPIC-Norfolk. Sucrose concentration in spot urine samples can be used as a concentration marker^(^ ^29^ ^)^ in order to rank participants according to intake. Given that we used spot urine samples to measure the biomarker, these samples required an adjustment for urine dilution. We used specific gravity, rather than urinary creatinine concentration, which is commonly used for this purpose. In our participants, urinary creatinine was highly significantly associated with BMI (*P*<0·0001) and therefore would have had a strong confounding effect on the observed association. In the previous analysis of EPIC-Norfolk, the ratio of urinary sucrose to fructose was used to adjust for the dilution effect in spot urine samples^(^ ^3^ ^)^. However, while this ratio was strongly associated with sugar intake and obesity risk, it is difficult to interpret as urinary fructose concentration depends not only on fructose but also on sucrose consumption, as sucrose is hydrolysed into glucose and fructose *in vivo*. In the present study we have therefore focused on urinary sucrose only and used urinary specific gravity to compensate for the dilution effect, although risk estimates remained similar when using sucrose:fructose as a measure of intake. While specific gravity is also associated with BMI, the association was weaker than for creatinine (*ρ*=0·08; *P*=0·0004). We could show that specific-gravity-adjusted urinary sucrose concentration was positively, and significantly, associated with self-reported intake from 7DD, 24HDR and FFQ.

Our results indicate that those in the highest category of sucrose intake as measured by the biomarker had the highest risk of being overweight or obese after three years of follow-up. Furthermore, we observed that a combination of low self-reported sucrose intake and high biomarker was associated with high BMI and WC. The tendency to under-report the intake of unhealthy foods and foods with high sugar content, especially among those who are overweight and obese^(^ ^9^ ^)^, may possibly be reason for the observed inverse association with self-reported intake. The positive association between BMI and the ratio of biomarker to self-reported intake suggests that participants with a higher BMI are more likely to under-report intake. As we have shown previously, the relationship between dietary and urinary sucrose is not affected by BMI^(^ ^15^ ^)^ and therefore does not explain this observation. There is some evidence suggesting that an important cause of under-reporting is the failure to report snack foods consumed between meals^(^ ^11^ ^)^ and biscuits, cakes, confectionery and other types of snacks were main contributors to total sucrose intake in EPIC-Norfolk (Table 4). A recent biomarker-based study detected substantial measurement error in self-reported sugar intake assessed by both FFQ and 24HDR, which was greater in women than men for both dietary assessment instruments^(^ ^16^ ^)^.

The strengths of the present study are the use of a biomarker as a surrogate measure of sucrose intake besides self-reported intake only, use of 7DD as a more detailed self-report dietary instrument and the well-characterised cohort. We also report findings for FFQ and 24HDR. However, there were also some limitations: sensitivity of our method for quantifying sucrose and fructose in urine was limited, which resulted in a reduced sample size and the pseudo-random selection of samples. Yet, analyses with imputed values for those outside the calibration range suggest that the reduced concentration range did not affect the observed associations. We also used spot urines, rather than 24 h urine collection, to measure sucrose and fructose. Furthermore, we report *P* values without adjustment for multiple testing and this must be taken into consideration when interpreting results. There were also some participants with gastric damage, which can lead to increased permeability of gastric mucosa for sucrose and increased excretion in urine^(^ ^30^ ^)^. However, gastric damage did not affect the outcome of our analyses.

There is some ambiguity in data from observational studies investigating associations between sugar intake and BMI. While sugar-sweetened beverage intake is clearly associated with an increased BMI^(^ ^4^ ^,^ ^5^ ^,^ ^31^ ^)^, the association is less clear for total sugar intake and sucrose and some studies report inverse associations. Indeed, recent guidance by the European Food Safety Authority^(^ ^7^ ^)^ suggests such an association. In the present study we observed a clear discrepancy between self-reported sucrose intake and biomarker-based findings in the relationship with BMI, despite correlation between the two measures of sugar intake. This is consistent with our previous results^(^ ^3^ ^)^. These data suggest that the inconsistency of data on sucrose and obesity may be in part attributed to misreporting and nutritional biomarkers are important to understand these associations better.

## Supplementary material

For supplementary material accompanying this paper visit http://dx.doi.org/10.1017/S1368980015000300. click here to view supplementary material
