# Supplementary material for: Association between sucrose intake and risk of overweight and obesity in a prospective sub-cohort of the European Prospective Investigation into Cancer in Norfolk (EPIC-Norfolk)
Source: Public Health Nutr. 2015 Feb 23;18(15):2815–24. doi: 10.1017/S1368980015000300 (PMC4595857; doi:10.1017/S1368980015000300)
Supplement: Supplementary file 1 [file S1368980015000300sup001.doc]

Supplementary material

Supplemental Table 1: Mean sucrose and fructose concentration and relative standard deviation of QC control samples. Samples were prepared by adding different amount of sucrose and fructose to pooled urine samples. Data shown were from at least ten repeat analyses.

|  | Extra Low | Low | | | Medium | High | Extra High^†^ |
| --- | --- | --- | --- | --- | --- | --- | --- |
|  | Sucrose | | | | | | |
| Mean [µM] | 5.2 | 6.9 | 11.3 | 22.0 | 50.3 | 102 | 422 |
| %CV | 6% | 10% | 10% | 11% | 10% | 20% | 16% |
|  | Fructose | | | | | | |
| Mean | 7.2 | 17.8 | 23.3 | 31.7 | 58.2 | 114 | 444 |
| %CV | 33% | 35% | 13% | 18% | 14% | 21% | 19% |

^†^analysed after dilution

Supplemental Table 2:

Baseline and second health check characteristics of 1734 participants of EPIC Norfolk for whom biomarkers were available. Participants were divided into quintiles of urinary sucrose biomarker and 7DD sucrose intake. Differences between respective quintiles were analysed using one-way ANOVA or χ^2^-tests.

|  | Q1 | Q2 | Q3 | Q4 | Q5 | Total | p |
| --- | --- | --- | --- | --- | --- | --- | --- |
| n | 347 | 347 | 347 | 347 | 346 | 1734 |  |
| Biomarker (urinary sucrose, adjusted by specific gravity) | | | | | | | |
| Range [µM/SG] | 5.0 – 11.1 | 11.2 – 21.1 | 21.2 – 39.9 | 40.0 – 80.2 | 80.3 - 493 | 5.0 – 493 |  |
| Age [year] | 58.5 (9.1) | 59.4 (9.0) | 58.7 (9.0) | 59.8 (9.0) | 61.1 (9.0) | 59.5 (9.1) | 0.0015 |
| BMI [kg/m2] | 25.7 (3.6) | 25.9 (3.6) | 26.1 (3.6) | 26.5 (3.6) | 26.8 (3.9) | 26.2 (3.7) | 0.0003 |
| BMI 2HC^¶^ [kg/m^2^] | 26.3 (3.8) | 26.6 (4.1) | 26.7 (3.7) | 27.1 (3.8) | 27.2 (4.0) | 26.8 (3.9) | 0.0130 |
| Waist circumference 2HC^¶^ [cm] | 86.3 (11.8) | 87.6 (12.4) | 88.7 (12.6) | 90.5 (12.7) | 91.5 (12.6) | 88.9 (12.6) | p<0.0001 |
| Energy intake [MJ/d] | 8.2 (2.0) | 8.1 (2.0) | 8.5 (2.1) | 8.5 (2.3) | 8.5 (2.4) | 8.4 (2.2) | 0.0177 |
| Sucrose intake [g/d] | 44.5 (21.8) | 47.8 (24.2) | 50.6 (25.0) | 54.8 (27.0) | 57.9 (28.5) | 51.1 (25.8) | 0.0012 |
| Contribution of sucrose to total sugars | 41% (11%) | 43% (10%) | 44% (10%) | 46% (11%) | 49% (12%) | 44% (11%) | P<0.0001 |
| Urinary sucrose [µM] | 7.6 (1.3) | 15.5 (1.2) | 30.0 (1.2) | 56.8 (1.2) | 156.0 (1.6) | 31.5 (2.9) | p<0.0001 |
| Sex |  |  |  |  |  |  | p<0.001 |
| Male | 120 (35%) | 144 (42%) | 165 (48%) | 173 (50%) | 195 (56%) | 797 (46%) |
| Female | 227 (65%) | 203 (59%) | 182 (52%) | 174 (50%) | 151 (44%) | 937 (54%) |
| Overweight^‡^ at 2HC^¶^ | 201 (58%) | 216 (62%) | 225 (65%) | 237 (68%) | 245 (71%) | 1124 (65%) | 0.004 |
| Sucrose intake (7DD, energy adjusted) | | | | | | | |
| Range [g/MJ/d] | 0.1 – 4.1 | 4.1 – 5.3 | 5.3 – 6.4 | 6.4 – 7.9 | 7.9 – 19.1 | 0.1 – 19.1 |  |
| Age [year] | 57.6 (8.9) | 59.2 (9.0) | 59.7 (9.0) | 60.5 (8.9) | 60.5 (9.1) | 59.5 (9.1) | p<0.0001 |
| BMI [kg/m2] | 27.3 (4.1) | 26.2 (3.7) | 26.3 (3.6) | 25.8 (3.5) | 25.4 (3.2) | 26.2 (3.7) | p<0.0001 |
| BMI 2HC^¶^ [kg/m^2^] | 27.9 (4.3) | 26.7 (3.8) | 26.8 (3.8) | 26.5 (3.7) | 26.0 (3.4) | 26.8 (3.9) | p<0.0001 |
| Waist circumference 2HC^¶^ [cm] | 93.4 (12.9) | 89.5 (12.5) | 89.6 (12.9) | 86.6 (11.7) | 85.4 (11.3) | 88.9 (12.6) | p<0.0001 |
| Energy intake [MJ/d] | 8.3 (2.2) | 8.6 (2.1) | 8.6 (2.3) | 8.1 (2.1) | 8.3 (2.1) | 8.4 (2.2) | 0.0077 |
| Sucrose intake [g/d] | 24.7 (9.6) | 39.0 (11.2) | 48.4 (13.1) | 59.9 (14.6) | 83.8 (27.1) | 51.1 (25.8) | p<0.0001 |
| Contribution of sucrose to total sugars | 33% (8%) | 40% (7%) | 44% (7%) | 48% (7%) | 57% (9%) | 44% (11%) | P<0.0001 |
| Urinary sucrose [µM] | 25.8 (2.7) | 31.8 (3.0) | 35.2 (2.9) | 33.4 (3.0) | 32.1 (3.0) | 31.5 (2.9) | 0.0022 |
| Sex |  |  |  |  |  |  | p<0.001 |
| Male | 195 (56%) | 184 (53%) | 174 (50%) | 125 (36%) | 119 (34%) | 797 (46%) |
| Female | 152 (44%) | 163 (47%) | 173 (50%) | 222 (64%) | 227 (66%) | 937 (54%) |
| Overweight^‡^ at 2HC^¶^ | 255 (73%) | 236 (68%) | 209 (60%) | 212 (61%) | 212 (61%) | 1124 (65%) | p<0.001 |

^¶^2^nd^ health check, three years after baseline; ^†^geometric mean and SD; ^‡^ BMI > 25 kg/m2

Supplemental Table 3:

Baseline and second health check characteristics of 797 male participants of EPIC Norfolk for whom biomarkers were available. Participants were divided into quintiles of urinary sucrose biomarker and 7DD sucrose intake. Differences between respective quintiles were analysed using one-way ANOVA or χ^2^-tests.

|  | Q1 | Q2 | Q3 | Q4 | Q5 | Total | p |
| --- | --- | --- | --- | --- | --- | --- | --- |
| N | 160 | 159 | 160 | 159 | 159 | 797 |  |
| Biomarker (urinary sucrose, adjusted by specific gravity) | | | | | | | |
| Range [µM/SG] | 5.0 - 13.3 | 13.4 - 26.2 | 26.2 - 49.5 | 49.7 - 99.4 | 99.4 - 472.1 | 5.0 - 472.1 |  |
| Age [year] | 58.7 (8.7) | 60.6 (9.1) | 59.8 (9.1) | 60.8 (9.2) | 62.6 (8.4) | 60.5 (9.0) | 0.0023 |
| BMI [kg/m2] | 26.3 (3.2) | 26.6 (2.9) | 26.4 (2.9) | 26.8 (3.4) | 26.5 (3.1) | 26.5 (3.1) | 0.6308 |
| BMI 2HC^¶^ [kg/m2] | 26.9 (3.5) | 27.0 (3.1) | 27.0 (3.3) | 27.3 (3.5) | 27.0 (3.3) | 27.0 (3.3) | 0.8771 |
| Waist circumference 2HC^¶^ [cm] | 96.3 (10.3) | 96.3 (8.5) | 96.6 (10.1) | 97.6 (10.2) | 97.6 (10.1) | 96.9 (9.9) | 0.5787 |
| Energy intake [MJ/d] | 9.5 (2.0) | 9.6 (1.9) | 9.5 (2.1) | 9.7 (2.2) | 9.7 (2.2) | 9.6 (2.1) | 0.7372 |
| Sucrose intake [g/d] | 49.3 (27.2) | 56.3 (28.1) | 57.2 (29.4) | 60.8 (27.6) | 68.1 (30.1) | 58.3 (29.1) | p<0.0001 |
| Contribution of sucrose to total sugars | 42% (13%) | 44% (12%) | 46% (11%) | 48% (12%) | 50% (12%) | 46% (12%) | p<0.0001 |
| Urinary sucrose [µM] | 8.7 (1.3) | 19.3 (1.2) | 36.3 (1.2) | 70.1 (1.2) | 169.8 (1.5) | 37.3 (2.9) | p<0.0001 |
| Overweight^‡^ at 2HC^¶^ | 105 (66%) | 114 (72%) | 115 (72%) | 119 (75%) | 120 (75%) | 573 (72%) | 0.308 |
| Sucrose intake (7DD, energy adjusted) | | | | | | | |
| Range [g/MJ/d] | 0.3 - 3.9 | 3.9 - 5.2 | 5.2 - 6.4 | 6.4 - 8.0 | 8.0 - 19.1 | 0.3 - 19.1 |  |
| Age [year] | 58.9 (8.5) | 60.5 (8.9) | 60.5 (8.9) | 60.9 (9.0) | 61.6 (9.4) | 60.5 (9.0) | 0.1001 |
| BMI [kg/m2] | 27.5 (3.3) | 26.8 (3.4) | 26.2 (2.6) | 26.1 (3.1) | 26.0 (2.8) | 26.5 (3.1) | p<0.0001 |
| BMI 2HC^¶^ [kg/m2] | 28.1 (3.6) | 27.4 (3.6) | 26.6 (2.8) | 26.5 (3.1) | 26.5 (3.1) | 27.0 (3.3) | p<0.0001 |
| Waist circumference 2HC^¶^ [cm] | 99.4 (10.1) | 97.9 (10.8) | 95.9 (8.8) | 95.6 (9.7) | 95.5 (9.3) | 96.9 (9.9) | 0.0005 |
| Energy intake [MJ/d] | 9.0 (2.0) | 9.4 (2.2) | 9.8 (2.1) | 9.8 (1.9) | 10.0 (2.0) | 9.6 (2.1) | 0.0001 |
| Sucrose intake [g/d] | 25.8 (9.7) | 42.7 (10.4) | 56.2 (12.7) | 69.4 (14.2) | 97.9 (26.1) | 58.3 (29.1) | p<0.0001 |
| Contribution of sucrose to total sugars | 32% (8%) | 41% (7%) | 45% (7%) | 51% (7%) | 61% (9%) | 46% (12%) | p<0.0001 |
| Urinary sucrose [µM] | 26.9 (2.7) | 31.5 (2.9) | 36.8 (2.6) | 44.3 (2.9) | 52.5 (3.0) | 37.3 (2.9) | p<0.0001 |
| Overweight^‡^ at 2HC^¶^ | 128 (80%) | 123 (77%) | 106 (66%) | 107 (67%) | 109 (69%) | 573 (72%) | 0.013 |

^¶^2^nd^ health check, three years after baseline; ^†^geometric mean and SD; ^‡^ BMI > 25 kg/m2

Supplemental Table 4:

Baseline and second health check characteristics of 937 female participants of EPIC Norfolk for whom biomarkers were available. Participants were divided into quintiles of urinary sucrose biomarker and 7DD sucrose intake. Differences between respective quintiles were analysed using one-way ANOVA or χ^2^-tests.

|  | Q1 | Q2 | Q3 | Q4 | Q5 | Total | p |
| --- | --- | --- | --- | --- | --- | --- | --- |
| n | 188 | 187 | 188 | 187 | 187 | 937 |  |
| Biomarker (urinary sucrose, adjusted by specific gravity) | | | | | | | |
| Range [µM/SG] | 5.0 - 9.6 | 9.6 - 17.6 | 17.7 - 35.8 | 35.9 - 67.4 | 67.7 - 492.5 | 5.0 - 492.5 |  |
| Age [year] | 57.9 (9.0) | 58.3 (9.0) | 58.8 (9.3) | 59.2 (8.9) | 59.0 (9.1) | 58.6 (9.0) | 0.6176 |
| BMI [kg/m2] | 25.4 (3.6) | 25.6 (4.4) | 25.3 (3.8) | 26.5 (4.0) | 26.6 (4.4) | 25.9 (4.1) | 0.0014 |
| BMI 2HC^¶^ [kg/m2] | 26.2 (3.7) | 26.3 (4.9) | 26.0 (4.1) | 27.2 (4.1) | 27.2 (4.4) | 26.6 (4.3) | 0.0118 |
| Waist circumference 2HC^¶^ [cm] | 81.4 (9.5) | 80.9 (10.5) | 81.0 (9.9) | 84.1 (11.0) | 83.3 (11.0) | 82.1 (10.5) | 0.0067 |
| Energy intake [MJ/d] | 7.4 (1.6) | 7.4 (1.6) | 7.3 (1.5) | 7.3 (1.7) | 7.2 (1.8) | 7.3 (1.6) | 0.6694 |
| Sucrose intake [g/d] | 41.5 (18.4) | 43.3 (18.4) | 44.1 (19.4) | 47.5 (22.5) | 48.6 (23.8) | 45.0 (20.8) | 0.0044 |
| Contribution of sucrose to total sugars | 40% (9%) | 42% (9%) | 43% (9%) | 44% (11%) | 46% (12%) | 43% (10%) | p<0.0001 |
| Urinary sucrose [µM] | 7.0 (1.2) | 13.2 (1.2) | 24.9 (1.2) | 47.8 (1.2) | 139.4 (1.7) | 27.3 (2.9) | p<0.0001 |
| Overweight^‡^ at 2HC^¶^ | 106 (56%) | 98 (52%) | 105 (56%) | 123 (66%) | 119 (64%) | 551 (59%) | 0.043 |
| Sucrose intake (7DD, energy adjusted) | | | | | | | |
| Range [g/MJ/d] | 0.1 - 4.3 | 4.3 - 5.3 | 5.3 - 6.4 | 6.4 - 7.8 | 7.8 - 16.5 | 0.1 - 16.5 |  |
| Age [year] | 57.7 (8.9) | 59.0 (8.8) | 58.5 (8.9) | 58.8 (9.4) | 59.3 (9.2) | 58.6 (9.0) | 0.4725 |
| BMI [kg/m2] | 26.7 (4.5) | 26.2 (4.3) | 26.1 (4.3) | 25.3 (3.6) | 25.2 (3.4) | 25.9 (4.1) | 0.0015 |
| BMI 2HC^¶^ [kg/m2] | 27.3 (4.7) | 26.9 (4.6) | 26.7 (4.4) | 26.0 (3.8) | 25.9 (3.5) | 26.6 (4.3) | 0.0048 |
| Waist circumference 2HC^¶^ [cm] | 84.2 (11.5) | 82.8 (10.9) | 81.9 (10.5) | 81.1 (10.0) | 80.8 (9.1) | 82.1 (10.5) | 0.0111 |
| Energy intake [MJ/d] | 7.0 (1.6) | 7.2 (1.6) | 7.3 (1.6) | 7.5 (1.5) | 7.5 (1.7) | 7.3 (1.6) | 0.0013 |
| Sucrose intake [g/d] | 22.9 (8.4) | 35.0 (8.2) | 43.0 (9.6) | 53.5 (11.6) | 70.7 (21.7) | 45.0 (20.8) | p<0.0001 |
| Contribution of sucrose to total sugars | 33% (8%) | 40% (7%) | 42% (6%) | 47% (7%) | 54% (9%) | 43% (10%) | p<0.0001 |
| Urinary sucrose [µM] | 22.1 (2.9) | 25.7 (2.9) | 26.7 (2.8) | 26.4 (2.8) | 37.9 (3.2) | 27.3 (2.9) | p<0.0001 |
| Overweight^‡^ at 2HC^¶^ | 128 (80%) | 123 (77%) | 106 (66%) | 107 (67%) | 109 (69%) | 573 (72%) | 0.013 |

^¶^2^nd^ health check, three years after baseline; ^†^geometric mean and SD; ^‡^ BMI > 25 kg/m2

Supplemental Table 5:
Association between sucrose intake and risk of being overweight or obese at baseline. Odds-ratio and 95% confidence interval determined by logistic regression, adjusted for age and sex.

|  | Men (n=797) | | Women (n=937) | | All (n=1734) | |
| --- | --- | --- | --- | --- | --- | --- |
|  | OR (95% CI) | p | OR (95% CI) | p | OR (95% CI) | p |
| Urinary sucrose - adjusted by specific gravity | | | | | | |
| Q1 | 1.00 | Ref | 1.00 | Ref | 1.00 | Ref |
| Q2 | 1.42 (0.89; 2.27) | 0.142 | 0.96 (0.64; 1.44) | 0.833 | 1.10 (0.81; 1.48) | 0.550 |
| Q3 | 1.21 (0.76; 1.91) | 0.423 | 1.02 (0.68; 1.54) | 0.918 | 1.32 (0.97; 1.79) | 0.077 |
| Q4 | 1.30 (0.82; 2.07) | 0.271 | 1.85 (1.22; 2.80) | 0.004 | 1.56 (1.15; 2.13) | 0.005 |
| Q5 | 1.12 (0.70 1.77) | 0.635 | 1.62 (1.08; 2.45) | 0.021 | 1.46 (1.07; 1.99) | 0.017 |
| Trend | 1.01 (0.91; 1.13) | 0.783 | 1.18 (1.08; 1.29) | 0.001 | 1.12 (1.04; 1.20) | 0.002 |
| Sucrose intake - 7DD, energy adjusted | | | | | | |
| Q1 | 1.00 | Ref | 1.00 | Ref | 1.00 | Ref |
| Q2 | 0.68 (0.41; 1.13) | 0.141 | 1.02 (0.68; 1.55) | 0.710 | 0.68 (0.50; 0.94) | 0.019 |
| Q3 | 0.44 (0.27; 0.72) | 0.001 | 0.69 (0.46; 1.04) | 0.146 | 0.57 (0.41; 0.76) | p<0.001 |
| Q4 | 0.46 (0.28; 0.75) | 0.002 | 0.64 (0.42; 0.96) | 0.068 | 0.51 (0.37; 0.70) | p<0.001 |
| Q5 | 0.50 (0.30; 0.81) | 0.005 | 0.57 (0.38; 0.87) | 0.076 | 0.48 (0.35; 0.66) | p<0.001 |
| Trend | 0.84 (0.76; 0.94) | 0.002 | 0.85 (0.78; 0.94) | 0.001 | 0.84 (0791; 0.90) | p<0.001 |

^†^2^nd^ health check BMI > 25 kg/m^2^

Supplemental Table 6:
Association between sucrose intake, determined using 7DD and different biomarkers, and risk of being overweight after three years of follow up. Odds-ratio and 95% confidence interval determined by logistic regression, adjusted for age and sex.

|  | Men (n=578) | | Women (n=660) | | All (n=1238) | |
| --- | --- | --- | --- | --- | --- | --- |
|  | OR (95% CI) | P | OR (95% CI) | P | OR (95% CI) | p |
| Urinary sucrose - adjusted by specific gravity | | | | | | |
| Q2 | 1.33 (0.76; 2.34) | 0.316 | 1.04 (0.64; 1.69) | 0.867 | 1.04 (0.72; 1.49) | 0.839 |
| Q3 | 1.38 (0.78; 2.44) | 0.267 | 1.20 (0.74; 1.95) | 0.464 | 1.63 (1.12; 2.37) | 0.010 |
| Q4 | 2.17 (1.19; 3.99) | 0.012 | 1.88 (1.14; 3.10) | 0.013 | 1.50 (1.03; 2.18) | 0.034 |
| Q5 | 1.38 (0.78; 2.45) | 0.268 | 1.64 (1.00; 2.69) | 0.050 | 1.68 (1.14; 2.46) | 0.008 |
| Trend | 1.12 (0.98; 1.28) | 0.090 | 1.17 (1.05; 1.31) | 0.005 | 1.15 (1.06; 1.26) | 0.001 |
| Sucrose intake - 7DD, energy adjusted | | | | | | |
| Q2 | 0.99 (0.53; 1.88) | 0.986 | 1.09 (0.67; 1.80) | 0.723 | 0.89 (0.60; 1.31) | 0.540 |
| Q3 | 0.59 (0.32; 1.08) | 0.086 | 0.94 (0.57; 1.54) | 0.800 | 0.65 (0.44; 0.95) | 0.026 |
| Q4 | 0.51 (0.28; 0.92) | 0.026 | 0.89 (0.54; 1.45) | 0.631 | 0.67 (0.46; 0.98) | 0.039 |
| Q5 | 0.64 (0.35; 1.17) | 0.149 | 0.75 (0.46; 1.22) | 0.243 | 0.63 (0.43; 0.93) | 0.019 |
| Trend | 0.86 (0.75; 0.98) | 0.024 | 0.92 (0.83; 1.03) | 0.156 | 0.89 (0.82; 0.97) | 0.006 |
| Urinary sucrose/fructose ratio | | | | | | |
| Q2 | 1.39 (0.79; 2.44) | 0.251 | 1.17 (0.72; 1.91) | 0.521 | 1.07 (0.75; 1.55) | 0.699 |
| Q3 | 1.22 (0.69; 2.14) | 0.488 | 1.02 (0.63; 1.66) | 0.931 | 1.16 (0.80; 1.68) | 0.434 |
| Q4 | 1.52 (0.86; 2.69) | 0.148 | 1.41 (0.86; 2.32) | 0.171 | 1.47 (1.01; 2.14) | 0.042 |
| Q5 | 2.17 (1.18; 4.01) | 0.013 | 1.76 (1.07; 2.91) | 0.026 | 1.72 (1.17; 2.54) | 0.006 |
| Trend | 1.17 (1.03; 1.34) | 0.018 | 1.14 (1.02; 1.27) | 0.021 | 1.15 (1.05; 1.25) | 0.002 |

Supplemental Table 7:
Association between sucrose intake (by biomarker or 7DD), BMI and waist circumference after three years of follow up at the 2^nd^ health check, adjusted additionally by physical activity. Regression coefficient β and 95% confidence interval determined by linear regression. Biomarker and intake data were log-transformed before analysis.

|  | Male (n=797) | | Female (n=937) | | |
| --- | --- | --- | --- | --- | --- |
|  | BMI at 2HC | | | | |
| Adjusted for | age | age + physical activity | | age | age + physical activity |
| Urinary sucrose (adjusted by specific gravity) | 0.09 (-0.13; 0.31) | 0.09 (-0.14; 0.31) | | 0.40 (0.14; 0.65) | 0.41 (0.16; 0.67) |
| Sucrose intake (7DD, adjusted by total energy) | -1.18 (-1.67; -0.69) | -1.18 (-1.68; -0.69) | | -1.60 (-2.25; -0.96) | -1.58 (-2.23; -0.93) |
|  | Waist circumference at 2HC | | | | |
| Adjusted for | age + height | age + height + physical activity | | age | age + height + physical activity |
| Urinary sucrose (adjusted by specific gravity) | 0.42 (-0.22; 1.06) | 0.40 (-0.24; 1.03) | | 0.85 (0.24; 1.46) | 0.90 (0.29; 1.52) |
| Sucrose intake (7DD, adjusted by total energy) | -3.35 (-4.78; -1.93) | -3.27 (-4.70; -1.85) | | -4.20 (-5.75; -2.64) | -4.06 (-5.61; -2.50) |

Supplemental Table 8:
Baseline and second health check characteristics of 3176 participants of EPIC Norfolk for whom all co-variables were available.

|  | Men | Women | All |
| --- | --- | --- | --- |
| N | 1297 | 1879 | 3176 |
| Range [µM/SG] | 4.8 - 492.7 | 4.8 - 495.1 | 4.8 - 495.1 |
| Age [year] | 60.2 (8.9) | 58.4 (8.8) | 59.1 (8.9) |
| BMI [kg/m^2^] | 26.4 (3.0) | 25.9 (4.0) | 26.1 (3.6) |
| BMI 2HC^¶^ [kg/m^2^] | 26.9 (3.3) | 26.5 (4.2) | 26.7 (3.9) |
| Waist circumference 2HC^¶^ [cm] | 96.3 (9.7) | 81.7 (10.5) | 87.7 (12.4) |
| Energy intake [MJ/d] | 9.6 (2.1) | 7.2 (1.6) | 8.2 (2.2) |
| Sucrose intake [g/d] | 57.4 (29.8) | 43.7 (20.3) | 49.3 (25.5) |
| Contribution of sucrose to total sugars | 45% (12%) | 42% (10%) | 44% (11%) |
| Urinary sucrose [µM] | 17.8 (3.8) | 12.1 (3.3) | 14.2 (3.6) |
| Overweight^‡^ at 2HC^¶^ | 128 (80%) | 123 (77%) | 106 (66%) |

^¶^ 2^nd^ health check three years after baseline; ^‡^BMI > 25 kg/m^2^

Supplemental Table 9:
Association between urinary sucrose biomarker^§^ and 7DD sucrose intake, BMI and waist circumference (at 2^nd^ health check) in all participants and in participants without self-reported gastric ulcer.

|  | Sucrose intake (7DD, energy adjusted, log-transformed)^&^ | | | BMI^&^ [kg/m^2^] | | | Waist circumference^¶^ [cm] | |
| --- | --- | --- | --- | --- | --- | --- | --- | --- |
|  | Men | Women | Sex-adjusted | Men | Women | Sex-adjusted | Men | Women |
| All | 0.10 (0.07; 0.13) | 0.06 (0.03; 0.08) | 0.08 (0.06; 0.10) | 0.09 (-0.13; 0.31) | 0.40 (0.14; 0.65) | 0.25 (0.08; 0.43) | 0.42 (-0.22; 1.06) | 0.85 (0.24; 1.46) |
| Without gastric ulcer | 0.10 (0.07; 0.13) | 0.06 (0.04; 0.09) | 0.08 (0.06; 0.10) | 0.08 (-0.15; 0.30) | 0.40 (0.14; 0.66) | 0.25 (0.08; 0.43) | 0.42 (-0.22; 1.07) | 0.88 (0.26; 1.51) |

^§^urinary sucrose adjusted by specific gravity;^&^ adjusted for age; ^¶^ adjusted for height and age

Supplemental Table 10:
Association between urinary sucrose biomarker and risk of being overweight or obese after three years of follow up in all participants and in participants without self-reported gastric ulcer. Odds-ratio and 95% confidence interval determined by logistic regression, adjusted for age.

|  | Men | | | | Women | | | | Sex-adjusted | | | |
| --- | --- | --- | --- | --- | --- | --- | --- | --- | --- | --- | --- | --- |
|  | All (n=797) | | Without gastric ulcer (n=760) | | All (n=937) | | Without gastric ulcer (n=907) | | All (n=1734) | | Without gastric ulcer (n=1667) | |
|  | OR (95% CI) | p | OR (95% CI) | p | OR (95% CI) | p | OR (95% CI) | p | OR (95% CI) | p | OR (95% CI) | p |
| Q2 | 1.31 (0.82; 2.11) | 0.263 | 1.44 (0.88; 2.35) | 0.147 | 0.84 (0.56; 1.27) | 0.416 | 0.81 (0.53; 1.22) | 0.306 | 1.15 (0.84; 1.56) | 0.384 | 1.14 (0.83; 1.56) | 0.419 |
| Q3 | 1.33 (0.83; 2.14) | 0.238 | 1.31 (0.81; 2.12) | 0.275 | 0.96 (0.64; 1.45) | 0.857 | 0.96 (0.63; 1.46) | 0.859 | 1.26 (0.92; 1.71) | 0.149 | 1.24 (0.91; 1.71) | 0.176 |
| Q4 | 1.54 (0.95; 2.50) | 0.082 | 1.44 (0.88; 2.35) | 0.146 | 1.45 (0.96; 2.21) | 0.080 | 1.38 (0.90; 2.11) | 0.142 | 1.43 (1.05; 1.96) | 0.025 | 1.46 (1.06; 2.01) | 0.021 |
| Q5 | 1.57 (0.96; 2.57) | 0.070 | 1.70 (1.03; 2.81) | 0.040 | 1.33 (0.88; 2.02) | 0.180 | 1.31 (0.86; 2.01) | 0.208 | 1.54 (1.12; 2.12) | 0.008 | 1.52 (1.10; 2.11) | 0.011 |
| Trend | 1.11 (1.00; 1.25) | 0.054 | 1.11 (0.99; 1.25) | 0.063 | 1.12 (1.02; 1.23) | 0.020 | 1.11 (1.01; 1.22) | 0.026 | 1.12 (1.04; 1.20) | 0.003 | 1.12 (1.04; 1.20) | 0.003 |
